# Marchiafava-Bignami disease: Case presentation and radiological imaging

**DOI:** 10.1016/j.radcr.2023.08.032

**Published:** 2023-08-26

**Authors:** Andrew Waack, Swamroop Nandwani, Meghana Ranabothu, Akash Ranabothu, Venkatramana Vattipally

**Affiliations:** a University of Toledo College of Medicine and Life Sciences, 3000 Arlington Ave, Toledo, OH 43614 United States; b Grand Valley State University College of Liberal Arts and Sciences, Mackinac Hall (MAK), 1 N Campus Dr, Allendale, MI 49401 United States; c Advanced Radiology Services, P.C.3264 North Evergreen Drive NE, Grand Rapids, MI 49525 United States

**Keywords:** Corpus callosum, Splenium, Centrum semiovale, Sandwich sign, Thiamine, Alcohol

## Abstract

Marchiafava-Bignami disease (MBD) is a rare vitamin B deficiency classically associated with alcoholism. MBD damages the corpus callosum and presents with nonspecific neurological symptoms. Radiological imaging is critical for diagnosing MBD and commencing subsequent treatment, which often consists of vitamin B supplementation. We present a case of MBD in a 56-year-old male with alcohol use disorder, epilepsy, schizophrenia, post-traumatic stress disorder, and cardiovascular risk factors. The patient presented with general neurological symptoms, and there were several potential diagnoses to consider based on the patient's history. Radiological imaging was necessary for diagnosis. This case demonstrates the role radiological imaging plays in the workup of MBD.

## Introduction

Marchiafava-Bignami disease (MBD) is a rare neurological condition that affects malnourished individuals and is typically due to chronic excessive alcohol consumption [1]. MBD causes a vitamin B deficiency that results in corpus callosum demyelination and necrosis [1,2]. MBD diagnosis is heavily dependent on radiological imaging and clinical history [3,4]. Prompt treatment is of paramount importance in achieving optimal outcomes, and the rate of favorable outcomes declines precipitously with delayed treatment [5]. Therefore, it is imperative for radiologists to recognize MBD and make a timely diagnosis to facilitate expedient treatment. We describe a case of MBD encountered in clinical practice and describe the pathophysiology, clinical presentation, imaging characteristics, and treatment strategies of MBD.

## Case presentation

A 56-year-old male with a prior medical history of chronic alcohol abuse disorder, schizophrenia, epilepsy, post-traumatic stress disorder, chronic anemia, hyperlipidemia, and hypertension presented to the emergency department (ED) for altered mental status. The patient was found alone at home with altered mentation and slurred speech by a family member. At presentation in the ED, the patient was hypertensive (blood pressure: 173/100) and tachycardic (123 beats per minute). Complete blood count revealed leukocytosis (white blood count: 15.21 x 10^3^; ref range: 4.5-11 x 10^3^ cells/mm^3^) and anemia (hemoglobin 12.9; ref range: 14-18 g/dL); comprehensive metabolic panel revealed hypernatremia (148; ref range: 135-145 mmol/L), hyperkalemia (5.2; ref range: 3.5-5 mmol/L), elevated creatinine (3.8; ref range: 0.7-1.3 mg/dL), blood urea nitrogen (53; ref range: 7-20 mg/dL), elevated aspartate aminotransferase (155; ref range: 12-38 U/L), and decreased glomerular filtration rate. Lactic acid levels were within normal limits. Urine toxicology and urinalysis were negative; urine drug screening was positive for cannabis. While in the ED, the patient had a short run of supraventricular tachycardia with a heart rate of over 170 beats per minute. The patient was treated with intravenous magnesium sulfate and subsequently admitted.

**Fig. 1:**
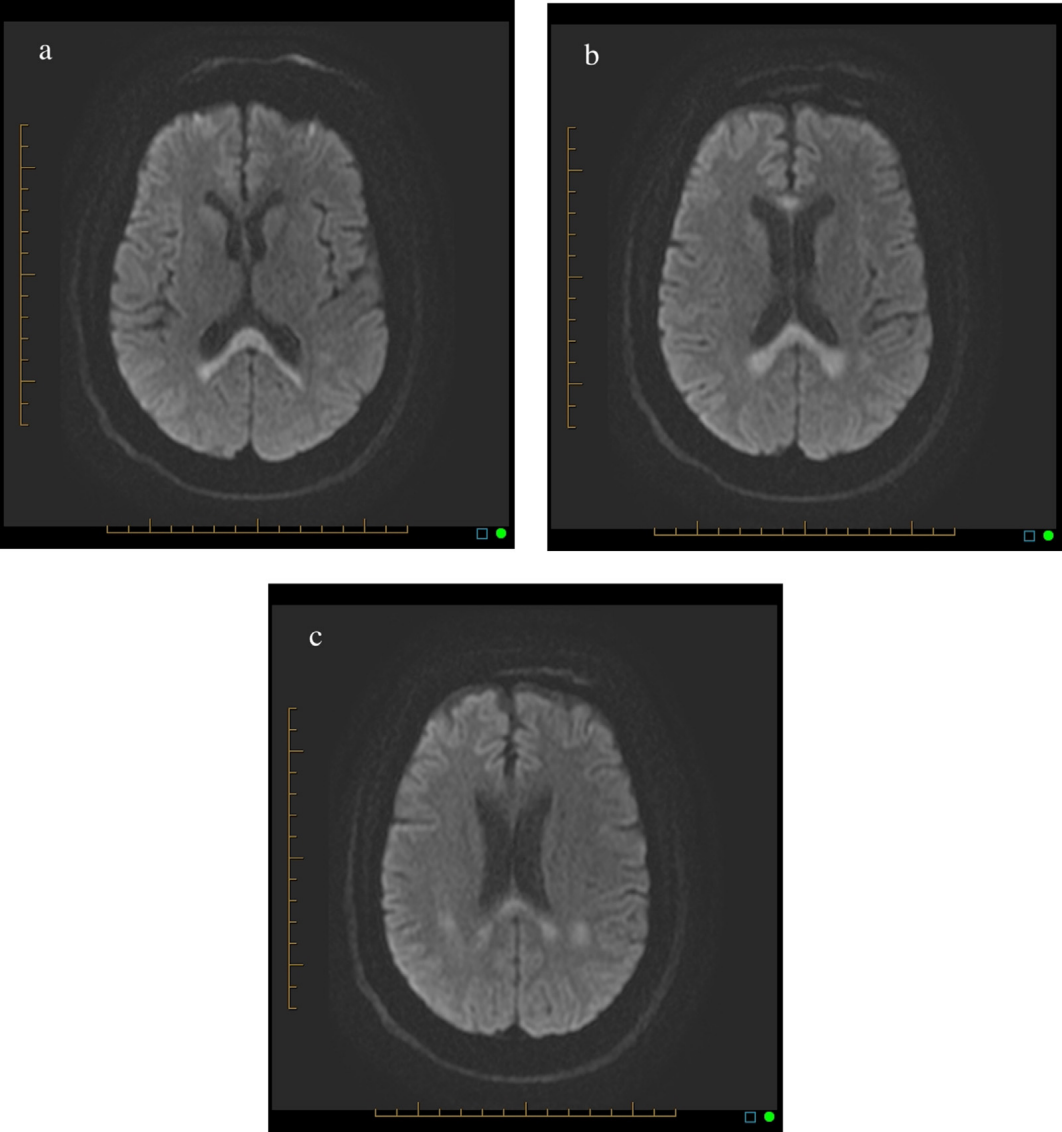
(A) Axial Diffusion weighted image demonstrating increased signal intensity in the splenium of the corpus callosum, representing restricted diffusion. (B) Axial Diffusion weighted image demonstrating increased signal intensity in the splenium and genu of the corpus callosum, representing restricted diffusion. (C). Axial Diffusion weighted image demonstrating increased signal intensity in the splenium of the corpus callosum and parietal centrum semiovale, representing restricted diffusion.

**Fig. 2:**
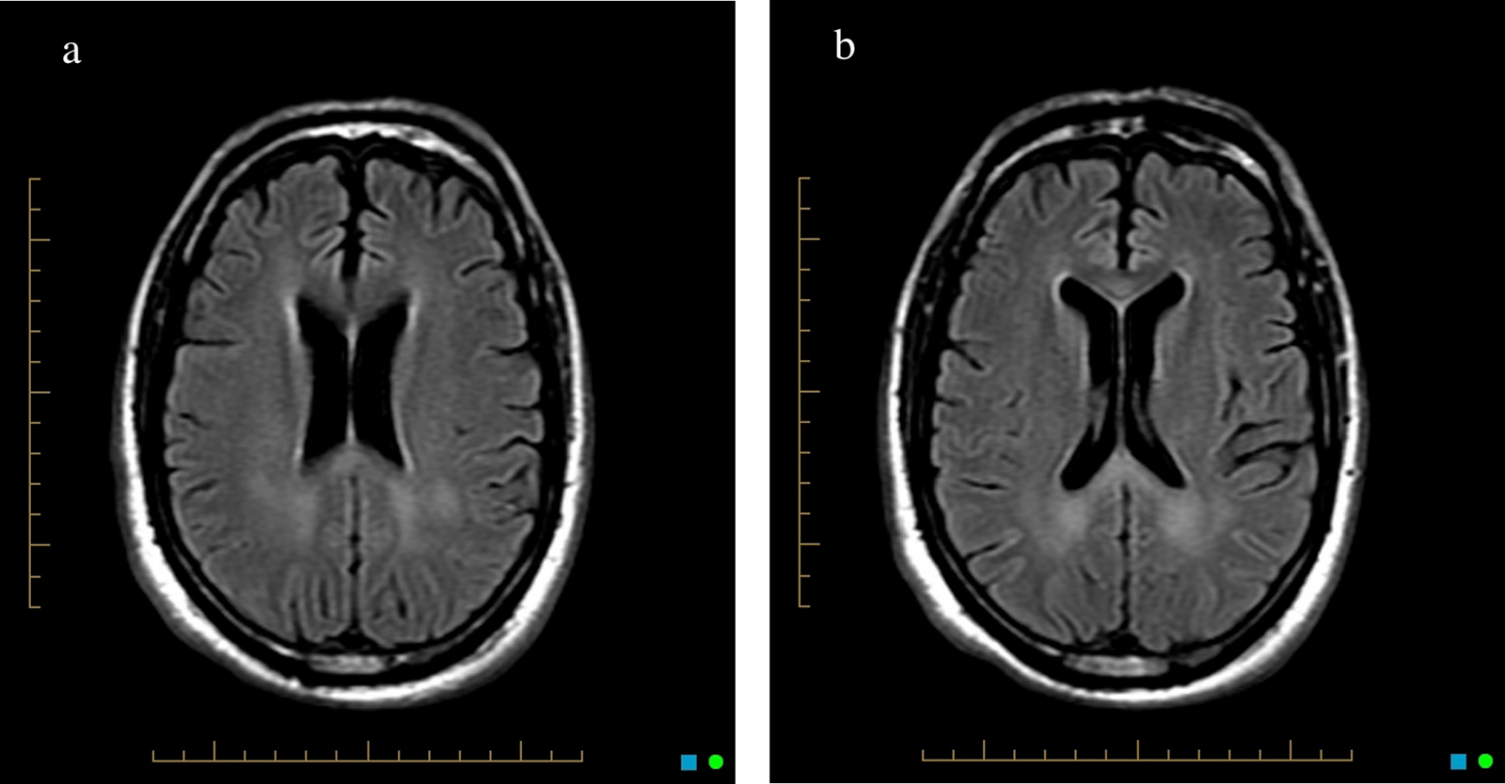
(A) Axial Fluid-attenuated inversion recovery image demonstrating increased signal intensity in the splenium of the corpus callosum and parietal centrum semiovale, correlating to the areas of restricted diffusion on diffusion weighted images (A–C). (B) Axial Fluid-attenuated inversion recovery image demonstrating increased signal intensity in the splenium and genu of the corpus callosum and parietal centrum semiovale, correlating to the areas of restricted diffusion on diffusion weighted images (A–C).

Computed tomographic (CT) imaging with contrast was unremarkable (not shown). Magnetic resonance imaging (MRI) with contrast revealed restricted diffusion of the genu and splenium of the corpus callosum, as well as the parietal centrum semiovale bilaterally (Figs. 1 and 2). Old basal ganglia lacunar infracts were seen bilaterally. There was minimal volume loss in the brain and small vessel ischemic disease in the periventricular and subcortical white matter. There was no evidence of abnormal signal in the mamillary bodies. All other intracranial findings were within normal limits. A diagnosis of MBD was made based on imaging findings and clinical history of chronic alcohol abuse.

Neurology and psychiatry were consulted during hospitalization. Psychiatry recommended increasing the patient's dose of risperidone, and neurology recommended an intravenous thiamine dosing regimen of 500 mg 3 times per day for 3 days, 250 mg 5 times per day for 5 days, and 100 mg daily chronically. The patient was also started on folic acid supplementation, magnesium oxide, and multivitamin and continued his existing medication regimen at the time of discharge.

## Discussion

MBD was first described by Marchiafava and Bignami in 1903 after observing multiple cases of seizures and coma occurring in middle-aged men who consumed excessive quantities of inexpensive Chianti wine; postmortem examination of these patients revealed corpus callosum degeneration [6]. This disease predominantly affects alcoholics, and it is most prevalent in men between 40 and 60 years old [1]. In a review of 153 reported cases of MBD, Hillbom et al. note that alcoholics comprise 92.8% of patients afflicted by MBD. Other conditions have rarely been reported to cause MBD in nonalcoholics, including depression hyporexia, bariatric and gastric surgery, and cerebral malaria [2,7,8].

The pathophysiology of MBD has not been fully elucidated, but it appears to be caused by vitamin B deficiency and the toxic effects of alcohol (although the disease can occur independent of alcohol consumption) [1,9]. Thiamine (vitamin B1) deficiency is especially important in MBD pathogenesis, and deficiency has been shown to alter cellular metabolism [2]. Toxic effects of alcohol include altered white matter protein expression, impaired lipid processing, decreased neural plasticity, and weakened vessels [2,10]. These insults culminate in small vessel necrosis, blood-brain barrier disruption, and cytotoxic edema, which manifests as symmetric demyelination and ischemic damage to the corpus callosum [2,7,9,10]. MBD is defined by corpus callosum destruction, but the destruction of other brain areas may occur in severe cases of MBD, including the deep cerebral white matter, optic chiasm and tracts, cortical gray matter, subcortical U fibers, cerebellar peduncles, and anterior commissure [2,4,11,12].

The clinical signs and symptoms of BMD are variable, and diagnosis requires a strong clinical suspicion based on patient history. The presentation relies heavily on the temporal course of disease: acute, subacute, and chronic presentations have been well described [1]. Acute BMD may present with decreased mentation, ataxia, delirium, dysarthria, coma, and death [2,4]. Subacute presentations often include confusion, memory deficits, gait impairments, behavioral alterations, and somnolence [2,4,12]. Chronic presentations include interhemispheric disconnection syndrome with sensory hemineglect and alien limb syndrome, progressive dementia, and aberrant behavior [2,4,7]. Patients can fully recover with complete resolution or progress to coma and eventual death [1,9].

Postmortem tissue examination was required for diagnosis prior to the advent of modern imaging techniques, but MBD can now be diagnosed radiologically [1]. On CT, MBD appears hypodense, unless there is associated hemorrhage, which causes an isodense or hyperdense appearance [1,4]. However, CT has poor sensitivity compared to MRI, and MRI is considered the “gold standard” imaging modality for MBD diagnosis [1]. Callosal lesions are hypointense on T1-weighted MR images, and hyperintense on T2, fluid-attenuated inversion recovery (FLAIR), and diffusion-weighted imaging (DWI) [9]. No mass effect is exerted, but extension beyond the callosal body into the genu and adjacent white matter is occasionally observed [9]. Acute MBD demonstrates a characteristic “sandwich sign,” in which the central corpus callosum body appears hyperintense on T2 and FLAIR MRI sequences, with relative sparing of the ventral and dorsal margins [1,2]. Chronic lesions may display well-defined cavitations [2]. In 2004, Heinrich et al. described 2 major clinicoradiological subtypes of MBD (Heinrich). Type A MBD is a subacute presentation with hypertonia, pyramidal signs, impaired consciousness, T2 callosal hyperintensity and an overall dismal prognosis; type B MBD presents with gait disturbance, interhemispheric disconnection syndrome, normal to slightly impaired mentation, T2 callosal hyperintensity and an overall good prognosis [3].

There are several differentials to rule out when considering a diagnosis of MBD. First, other encephalopathies classically associated with chronic alcoholism must be considered, including Wernicke's encephalopathy, Korsakoff syndrome, and Wernicke-Korsakoff syndrome; the clinical presentation differs from MBD, as Wernicke's typically includes ophthalmoplegia, nystagmus, and ataxia [1]. Other differentials include demyelinating disorders, such as multiple sclerosis, progressive multifocal leukoencephalopathy, and acute disseminated encephalomyelitis (Singh); recurrent artery of Heubner infarction and central pontine myelinolysis should also be considered as well [4,12].

There are currently no established treatment guidelines for MBD due to the condition's rarity, and existing literature describing MBD is limited to case reports, case series and reviews [2]. Previously reported treatment strategies include vitamin B administration, corticosteroids, and amantadine. Intuitively, MBD often improves following the administration of vitamin B and can lead to full recovery [2]; however, the treatment must be rapidly initiated: Hillbom et al. report superior results if parenteral thiamine is administered within 2 weeks of symptom onset [5,7]. Several authors have reported the benefit of the administration of corticosteroids, possibly by reducing vasogenic edema [1,2,11]. However, corticosteroids are not necessary if thiamine is administered, and Hillbom et al. state that corticosteroids confer no benefit [2,5]. Lastly, amantadine has been used by several authors for treating MBD [[13], [14]–15]. It is not known exactly how this drug treats MBD, but it is hypothesized to modulate affected dopaminergic pathways [13]. Notably, in all reported cases, amantadine was used in conjunction with vitamin B supplementation, so it is not clear if amantadine provides benefits in MBD [13]. Lastly, seizures, coma, and associated symptoms can be treated symptomatically [1]. There are several potential courses of disease, including persistent symptomatology, progression to a vegetative state, and subsequent death, or recovery [2]. Over half of nonalcoholic MBD patients make full recoveries, while only 10% of alcoholic MBD patients recover fully [2]. Alcohol cessation, rehabilitation, and nutritional counseling are recommended in alcoholic patients who do survive [1,12].

## Conclusion

MBD is a rare neurological condition resulting from malnourishment with insufficient vitamin B levels. The overall prognosis is poor, but the best outcomes result from prompt diagnosis and treatment initiation [5]. MBD is clinicoradiological diagnosis [3]. MBD is defined by T1 hypointensity and T2, FLAIR, DWI hyperintensity within the corpus callosum [1,2,13]. It is important for radiologists to consider MBD in their list of differentials to achieve optimal outcomes.

## Patient consent

Patient consent obtained.
